# Dynamic Eye Tracking Based Metrics for Infant Gaze Patterns in the Face-Distractor Competition Paradigm

**DOI:** 10.1371/journal.pone.0097299

**Published:** 2014-05-20

**Authors:** Eero Ahtola, Susanna Stjerna, Santeri Yrttiaho, Charles A. Nelson, Jukka M. Leppänen, Sampsa Vanhatalo

**Affiliations:** 1 Department of Children’s Clinical Neurophysiology, Helsinki University Central Hospital, Helsinki, Finland; 2 Department of Biomedical Engineering and Computational Science, Aalto University, Helsinki, Finland; 3 Infant Cognition laboratory, Tampere Center for Child Health Research, University of Tampere, Tampere, Finland; 4 Laboratories of Cognitive Neuroscience, Children's Hospital Boston & Harvard Medical School, Boston, Massachusetts, United States of America; 5 Department of Neurological Sciences, University of Helsinki, Helsinki, Finland; UNC Chapel Hill, United States of America

## Abstract

**Objective:**

To develop new standardized eye tracking based measures and metrics for infants’ gaze dynamics in the face-distractor competition paradigm.

**Method:**

Eye tracking data were collected from two samples of healthy 7-month-old (total n = 45), as well as one sample of 5-month-old infants (n = 22) in a paradigm with a picture of a face or a non-face pattern as a central stimulus, and a geometric shape as a lateral stimulus. The data were analyzed by using conventional measures of infants’ initial disengagement from the central to the lateral stimulus (i.e., saccadic reaction time and probability) and, additionally, novel measures reflecting infants gaze dynamics after the initial disengagement (i.e., cumulative allocation of attention to the central vs. peripheral stimulus).

**Results:**

The results showed that the initial saccade away from the centrally presented stimulus is followed by a rapid re-engagement of attention with the central stimulus, leading to cumulative preference for the central stimulus over the lateral stimulus over time. This pattern tended to be stronger for salient facial expressions as compared to non-face patterns, was replicable across two independent samples of 7-month-old infants, and differentiated between 7 and 5 month-old infants.

**Conclusion:**

The results suggest that eye tracking based assessments of infants’ cumulative preference for faces over time can be readily parameterized and standardized, and may provide valuable techniques for future studies examining normative developmental changes in preference for social signals.

**Significance:**

Standardized measures of early developing face preferences may have potential to become surrogate biomarkers of neurocognitive and social development.

## Introduction

The emergence of core attention processes (e.g., visuospatial orienting) and preferential attention to social cues (e.g., faces) during the first postnatal months provide foundations for cognitive and social brain networks, and may be critical in initiating the developmental process that leads to a full repertoire of human social skills [1]. In recent year, there has been increasing interest in charting the typical developmental time course of these processes in human infants [2]–[4], and in deviations from the typical trajectory as a potential marker of certain neurodevelopmental disorders [5].

The endeavors to characterize the early development of attention and face preferences in infants are critically dependent on methods that i) can be successfully implemented with poorly co-operating infants of various postnatal ages, ii) enable standardized and, preferably, automated acquisition of metrics for the cognitive processes of interest, and iii) will eventually allow sufficient norms to be collected for the measures of interest to effectively characterize the performance of individual infants. In this context, it is interesting to note that recent development of semi- or fully automated eye tracking systems based on infrared reflections from the cornea has provided laboratories with more objective indices of infant gaze behavior [6], and that these methods have been successfully used to measure infants’ visuospatial orienting [3], [4], [7], [8] and attention to the eyes [5] or faces [2] in complex social scenes.

In the present study, our purpose was to further develop eye tracking based assessment of infant cognition in the context of the classic overlap paradigm [9]. Studies using this paradigm have typically examined the latency and/or frequency of infants gaze shift from the first (centrally presented) stimulus to the second (lateral) stimulus, putatively reflecting the active process of attention disengagement and visuospatial orienting. These processes undergo rapid developmental improvements during the first months of life and reach apparent stability at around six months of age [3], [4], [10], [11]. Sensitivity to faces and facial expressions in the overlap paradigm emerges at 5 and 7 months of age and is manifested as a delayed latency and reduced probability of gaze shifts in the context of faces, particularly when the face displays a fearful expression [12]–[14].

Infant gaze shifts in the overlap paradigm have been analyzed by using manual scoring of eye movements from video records [14], by using electro-oculogram [15], and more recently, by using eye tracking [3], [8], [16]. Compared to other, mostly manual techniques, eye tracking has the advantage of offering the possibility for completely automated acquisition and analysis of eye movements at a high spatial and temporal resolution. However, the practice of such analyses is still complicated by several limitations that surround current eye tracking technologies and affect data quality [16]–[20], and may require several verification routines to be implemented successfully in poorly co-operating participants [21].

Our goal in the present study was to further develop the eye tracking based assessment of infants gaze behavior in the overlap paradigm. First, our objective was to implement the eye tracking testing in a clinical environment by using automated testing and data analysis protocols. Second, we extended previous analyses focusing on infants’ first gaze shifts [9], [13] by exploring the dynamics of infants gaze movements over a longer time period, covering the entire trial time. Evidence from a previous study [22] shows that infant’s initial gaze shift from a face stimulus to a lateral distractor is routinely followed by a quick re-engagement of attention with the face, and that the latency of this re-engagement is modulated by facial expression. Other prior studies have suggested that cumulative preference for social over non-social stimuli [23], as well as the tendency to look back at a person after momentary distraction [24] may reflect important aspect of early social development. Taken together, these lead to prediction that eye tracking based parameterization of this phenomenon may provide useful metrics for the assessment of infants’ face preferences.

Given that our the analyses beyond the first gaze shift were highly explorative in nature, we examined whether the results obtained from the analysis of our primary data from 7-month-old infants could be replicated in an independent sample of infants of the same age, and whether the hereby introduced parameter would be sensitive to the known developmental difference in face preference between 7- and 5-month-old infants [2], [25]. These analyses were important for testing the feasibility of the new metrics in future, larger scale studies to establish normative data on the early development of visual attention and face preferences, and the potential use of such norms to detect deviations from the typical developmental trajectory in early infancy.

## Materials and Methods

### Participants

**Figure 1:**
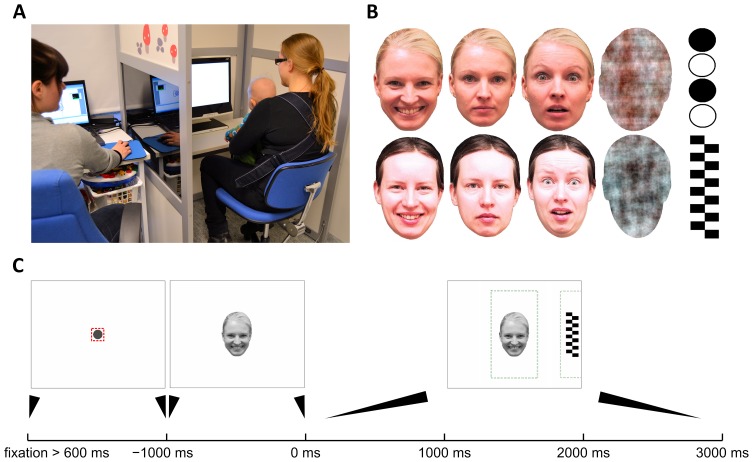
Eye tracking setup and the paradigm. A) The photograph shows the whole study setup where infant is placed into a baby carrier attached to the chest of the caregiver (right), and the experimenter (left) is sitting behind a light wall with one-way transparency (not visible on the photograph) to allow observation of the infant during test protocol. One-way transparency was created by tinting the window and having higher lighting level on baby’s side. B) The two sets of face stimuli used in our study. Every infant was presented stimuli from one set only (chosen randomly). Both sets included a face with happy, neutral, and fearful expressions, as well as a noise (or ‘non-face’) stimulus that was created by a phase-scrambling of a face image to retain many physical image properties. Peripheral distractors (called ‘target’) consisted of checkerboards and other geometrically simple high-contrast stimuli. C) Content of a trial. Each trial was preceded by showing a fixation cue in the center of the screen to attract infant’s attention. The trial started only after infant’s fixation in the central area (small square around the dot) had lasted 600 ms. Then, the face stimulus was shown for 1000 ms, followed by adding the target to either side of the screen for the remaining 3000 ms. The stippled lines depict areas of interest (AOI) used for computing MD and DT metrics. The AOIs were deliberately defined to be larger than the original stimuli to cancel out effects of measurement inaccuracies and to reduce unnecessary noise in DR traces (see also figure 2 and Discussion on spatial accuracy in Supplementary data).

The primary sample consisted of 13 infants tested at the Helsinki University Hospital (N = 13; 10 females; age range 7.1–8.0 months, mean = 7.50). The data from an additional three infants were excluded from the analyses due to technical difficulties in eye tracking (n = 2) or medication (n = 1). To test the metrics developed in the analyses of the primary sample, we analyzed data from an additional sample of 7-month-old and 5-month old infants (total n = 54); for a detailed description, see Dataset S1). All infants were born full term and reported to have a typical development. The Ethics Committee of the Hospital District of Helsinki and Uusimaa approved the study protocol in Helsinki, and the Boston Children's Hospital Committee on Clinical Investigation approved the study in Boston. The parents signed an informed consent. The individual shown in figure 1 of this manuscript has given written informed consent (as outlined in PLOS consent form) to publish her photograph.

### Test procedure

Infants were placed sitting in a baby carrier attached on their parent’s chest, and a sequence of visual stimuli were presented on 17-inch TFT monitor integrated in an eye tracker device. The ambient light was kept dim, and the participants (baby and caregiver) were separated from the experimenter by light walls (see figure 1A). Before running the actual study protocol, a calibration procedure was performed as explained in detail in the Supplementary data (information S1 and figure S1).

Each trial consisted of two phases, which together lasted for 4000 ms (figure 1C). The trial began by first attracting child’s attention to the center of the screen using simple audiovisual animations, for example a gradually expanding red circle (diameter from 0.3° to 4.2°) with recurring sound. The trial was programmed to start automatically only after the eye tracking device had reported 600 ms of continuous fixation onto the predefined ‘fixation area’ (diameter 4.2°) around the animation stimulus (see figure 1C) resulting in variable interstimulus interval between successive trials (M = 3090 ms, SD = 590 ms). During the first 1000 ms, a face image was shown on the center of the screen. During the remaining 3000 ms, a peripheral stimulus (hereafter called “target”) was added into the edge of the screen 10.2° away from the face, equiprobably on the left or right. The trials (32 altogether) alternated randomly between four different face images, each followed by a peripheral target. Each face stimulus was presented eight times with the only constraints being that the same face was presented no more than twice in a row, and the target was no more than three times in a row shown on the same side of the screen. An example video clip of typical progression of consecutive trials is presented among the Supplementary data (video S1).

### Stimuli

We used two sets of face images (figure 1B) copied from prior studies [26], each consisting of three different facial expressions (i.e. a colour image of a female model with neutral, happy, or fearful facial expression) and a sham face (i.e. phase-scrambled face that retained the amplitude and colour spectra as well as the contour of the face stimulus but was not identifiable as face). At the average viewing distance of 55 cm, the face stimuli covered visual angle measuring 8.2° horizontally and 11.7° vertically. The target stimuli were black-and-white vertically arranged circles or a checkerboard pattern, measuring 11.2° vertically and 3.2° horizontally.

All stimuli were presented electronically using E-Prime software (version 2.0.8.22, Psychology Software Tools, Pittsburgh, PA) and E-Prime Extensions for Tobii (version 2.0.1.5), interfacing with the eye tracker hardware.

### Eye tracking system

The eye tracker system used in our study was Tobii T120 (Tobii Technology AB, Stockholm, Sweden) that is equipped with an integrated 17” TFT display (refresh rate: 60 Hz, response time: 4 ms, screen resolution: 1280×1024 pixels). The system samples eye tracking data at 120 Hz (i.e. temporal accuracy is 8.3 ms), operates at 50–80 cm distances from the eyes, and can follow head movements within a window of 30×22cm (at 70 cm from the screen). According to the manufacturer, this system has a spatial accuracy in the order of 0.5 degrees, which corresponds to 4.4–6.9 mm on screen at the allowed viewing distance. In order to verify these accuracy measures in our present context, we ran additional testing of spatial accuracy as explained in detail in the Supplementary data (information S2 and figure S2).

Tobii eye tracker system is based on Pupil Centre Corneal Reflection (PCCR), that is, near infrared illumination and its reflections from the cornea relative to the center of the pupil. The light reflections are captured by two cameras and a general 3D model of the eye and the angles, distances and other geometrical features of the reflections are used to calculate the positions of the eyes and the direction of gaze.

### Data Analysis

Our present work proceeded in four overlapping and partly reiterating steps. First, we replicated previous studies by examining the latency and probability of gaze shifts from the face to the lateral distractor. Second, we extended these analyses by investigating time varying changes in gaze after the initial disengagement from the face stimulus in the context of different facial stimuli. This analysis was performed in a completely data-driven fashion, with the only preset criteria being the exclusion of trials with technically unreliable tracking or an absence of gaze shift from the face to peripheral target (see below). Third, we assessed different strategies in parameterizing the time-varying changes in attention beyond the initial disengagement. Fourth, we examined the replicability of the metrics extracted from the primary sample (Helsinki) in an independent sample of 7-month-old infants (Boston, 7-month sample), and the sensitivity of the metrics to developmental differences by comparing the primary data with data collected from 5-month-old infants (Boston, 5-month sample).

All data analysis was performed offline in MATLAB environment (version R2010a, The MathWorks, Natick, MA) in a way that will allow later straightforward automatization of the analytic procedure.

#### Structure of the raw data

The eye tracking data was written into an ASCII file that contains multiple time series sampled at 120 Hz. These series include 1) x- and y-coordinates for the point of gaze on the screen, 2) timestamps for each data sample at microsecond accuracy, 3) ‘validity codes’ for each eye indicating the reliability of tracking at each time point (out of codes 0–4 we only used time samples with validity code 0 or 1, which were taken to indicate technically reliable gaze tracking), and 4) additional index that characterizes the exact timing of changes in stimulus presentation.

#### Areas of interest (AOI) and AOI-transformed gaze data

**Figure 2:**
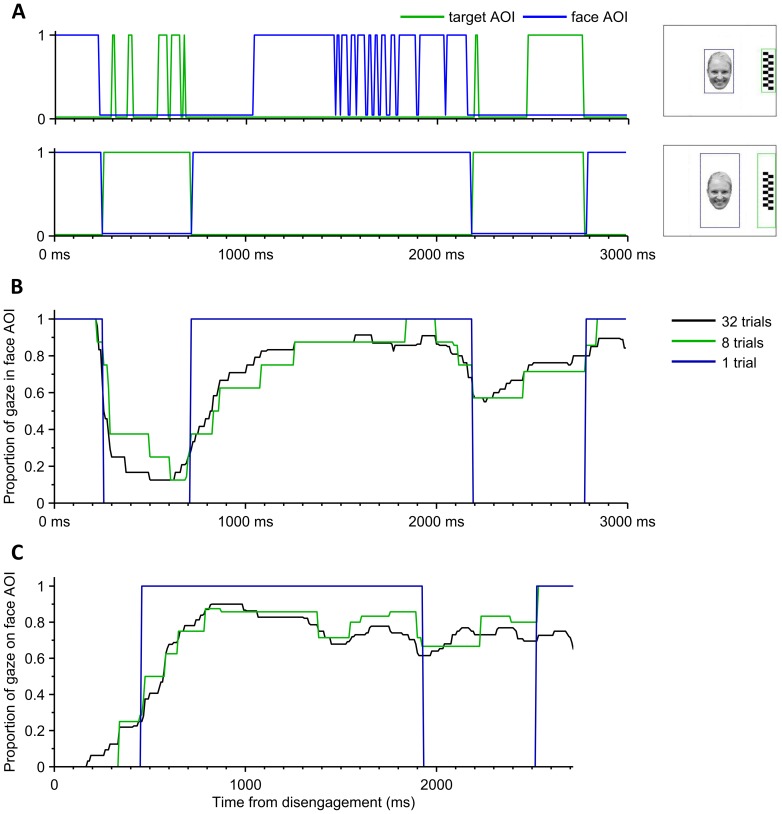
Nature of the eye tracking data. A) These two graphs demonstrate how widening AOIs (shown in the right hand side) affect the binary time series computed from the same eye tracking data of one single trial. using Widening the additional horizontal margins of face AOI from 0° (top graph) to 3.4° (120 pixels; bottom graph) removes the spiky appearance in the face and target AOIs (blue and green, respectively). This we consider as “measurement noise” due to many uncontrollable factors, and we reason that the dynamically more stable AOI time series with wider AOI margins would better reflect the cognitive phenomena of our interest. For instance, infant’s gaze is first directed to the face, but switches to peripheral target for a period between 250 and 700 ms translating into the disengagement time (DT) of 250 ms. If narrower margins were applied (top), a slightly delayed DT would be measured, and the dynamics of the last 2000 ms in trial would be highly distorted. B) A representative example of a dynamic average responses of one participant with different number of trials taken into the averaging (1, 8, and 32). The response resolution (step size) in vertical axis is always inversely proportional to the number of trials included in the averaging. C) An example of disengagement time-locked dynamic responses. Prior to averaging, each trial time series is shifted in time according to the moment when gaze first reached the target AOI (DT time-locking) and the data before that is omitted. This approach offers better representation of gaze changes where DT may be variable across trials. Note that the data presented here is from the same experiment as in figures A and B.

Our analysis was based on quantifying the amount of time that the infant gazed at the face or the target. To this end, we defined areas of interest (AOI) around the stimuli (see also figure 2A) and converted the 2D gaze tracking data coordinates into binary streams for each AOI (1 = gaze within the AOI; 0 = gaze outside of AOI). No fixation filter was applied on the raw point of regard data. After inspection of a considerable amount of full time series of gaze data, we concluded that only negligible amount of gaze data would fall outside the face or the target. This allowed us to deliberately widen the margins of AOIs around face and target (by 3.4° visual angle, see figure 2A), leading to a substantial reduction of measurement noise that comes from inaccuracies in eye tracking near AOI margins especially with infants with suboptimal calibration. Figure 2A shows an example of the AOI-transformed data in one trial and the effect of the size of the AOIs on the AOI time-series.

#### Gaze disengagement time (DT) and probability (DP)

Disengagement time (DT) and probability (DP) are the conventional measures in this test paradigm [25], [26]. DT is defined as the latency from the target onset to the time when the infant shifts gaze from the face to the target. DP is the proportion of gaze shifts out of all accepted trials (i.e., trials with a gaze shift and trials on which the gaze shift was not observed within 1000 ms after the onset of the peripheral stimulus). Consistent with prior studies, all infants with ≥2 scorable DTs per condition were retained in the DT analysis (N = 10, range 2–8, Means 4.0–6.1) and all infants with ≥3 scorable trials per condition in the DP analysis (N = 13, range 6–8 trials, Means 7.7–7.9).

#### Time-varying characteristics of gaze after initial disengagement – the novel dynamic response (DR)

In order to evaluate the dynamics of infants’ gaze after initial disengagement, we calculated the mean responses for each subject from AOI-transformed binary time series of all accepted time points in all trials. In this analysis, data from each trial was first co-aligned in time according to the moment when gaze first reached the lateral distractor AOI (i.e., post disengagement). The resulting time series constitutes the dynamic response (DR) reflecting the time-varying characteristic of infant gaze between the distractor and face AOIs. The averaging for each participant was performed either separately for every stimulus to produce four stimulus-specific responses (happy, neutral, fear, and phase-scrambled face) or jointly for all face stimuli yielding one face response averaged from larger (up to n = 24; phase-scrambled face excluded) number of trials. We omitted trials where gaze shift (face to target) did not happen (36.9% of all trials with face stimuli; more details in table S1). The trial retention rate is similar to that in previous studies using the same paradigm with typically developing children and children with developmental disorders (e.g. [27]). The averaged time series were further median filtered (with a window of 15 samples, equals 125 ms in time) in order to moderate abrupt, mostly technical artefacts (spikes and drops; see figure 2A). Consequently, the DR results are robust against noisy data or technically missing (invalid) data points.

In the DR traces, the value at each time point represents the estimated probability of the gaze being at the face AOI. For example, a probability value of 0.8 in the average response curve means that the infant’s gaze was directed to the face in 80% of the trials at this given moment. Here, the “vertical” step size (resolution) of the response is inversely proportional to the number of trials taken into the average (figure 2B).With the maximum of 24 trials (from all face stimuli) the step size would be 0.042 units of probability.

#### Computation of Mean Deviance (MD)

**Figure 3:**
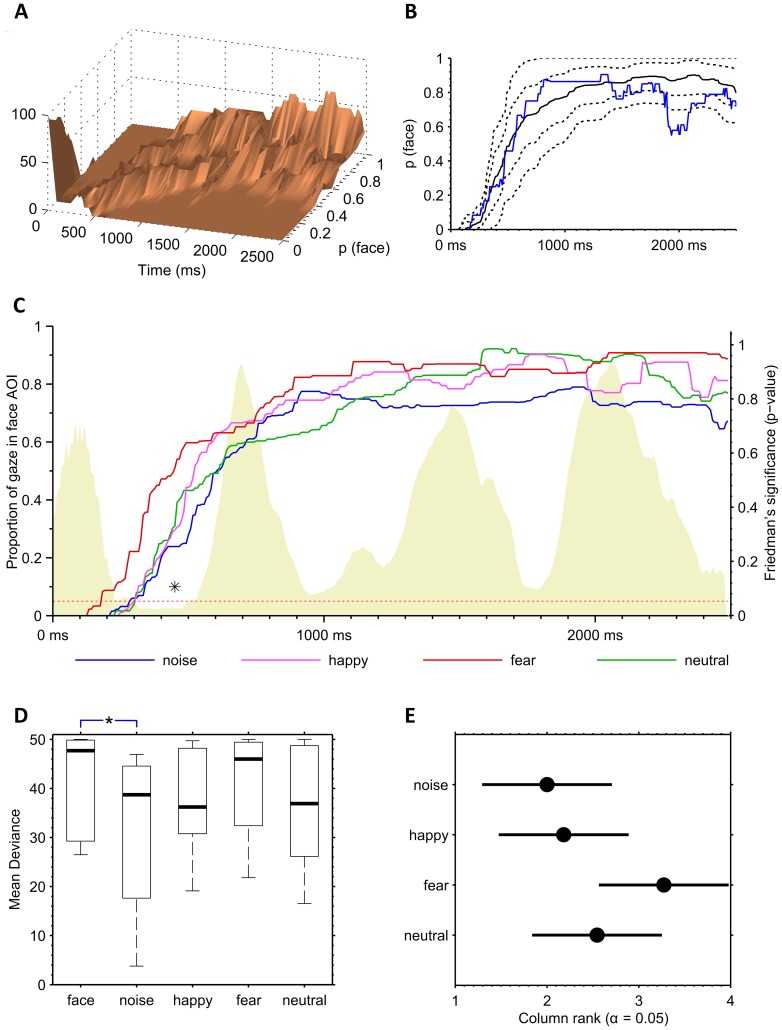
Statistical characteristics of the eye tracking data. A–B) Properties of the DT time-locked face AOI dynamic response (DR). The summary graph of DR average responses over all individuals (A) reveals clear changes of distribution within the trial time. These distributions were used in the graph B where we computed bootstrapped estimates (5000 samples) of the grand median DR (black solid line) as well as the 10th, 25th, 75th, and 90th percentile ranges (black stippled lines). The percentiles used in computation of the mean deviance (MD metric) of an individual DR trace (blue) were however from the original DR data of the control group, not from the bootstrapped distribution. C) DT time-locked average dynamic responses (DR) for each stimulus condition. The non-face stimulus (blue) yielded generally lower DR values, and the DR trace after fearful face (red) was generally higher. There were only marginal differences if the traces were compared for each time point. The smoothed time-series of p-values of non-parametric Friedman’s tests (shaded area, right side vertical axis) computed for each time point (8-ms time frame) reveals one local minimum that yields p-values under 0.05.This occurs between 350 and 500 ms from the disengagement which is a typical time frame for the gaze to return from the distractor. D) Comparison of stimulus-specific Mean Deviances (MD) derived from the individual DR. There was a significantly lower MD with non-face stimulus (noise) compared to combined face condition (p = 0.05; Mann-Whitney U test). MDs were computed from the 13 infants of the Helsinki control group and are presented in the boxplot graphs on the left. E) Post-hoc analysis (Tukey’s test) comparing MDs of the stimulus conditions. Each condition is represented by the group mean rank (circle) and the associated confidence interval. No significant differences are found. Lack of significant differences between facial expressions was probably due to the limited numbers of i) subjects in comparison and ii) trials inside MDs (max 8).

For the dynamic response (DR), we devised a measure of mean deviance that quantifies how much participant’s DR (when locked to the DT-time) deviates from the normative DR responses during the given time window. This is expressed as the average (in time) displacement of DR under the primary sample median DR expressed in percentiles of the DR distribution in primary sample (figure 3B). The best matching percentile for each DR value was calculated by interpolating the primary sample distribution (figure 3A). Because we were especially interested in downward deviations all DR values above the 50th percentile (median) would count as 50 in the averaging. Such score is a measure of mean deviance’ (MD), and it will give 50 if the participant’s response is entirely within the upper 50 percentiles whereas values decreasing below 50, in turn, would imply increasing deviations from the normative range. Figure 3F demonstrates the difference between group median response and 90th, 75th, 25th, and 10th percentiles, as well as an example of a DR of an individual infant.

#### Statistical procedures

The first set of statistical analyses examined how the conventional dependent variables (DT and DP) were affected by the facial expression condition, using data from the primary sample. Different facial expression conditions were compared by using Friedman test, followed by post-hoc testing with Tukey’s test. Bonferroni correction was used in multiple comparisons when appropriate. Test was considered significant if p<0.05. Second, we examined whether the MD varied as a function of facial expression by using similar statistical tests as those used in the analysis of DT and DP. We also examined whether the distribution of MD scores obtained from the primary data could be used to identify i) a new sample of 7-month-old infants as similar (suggesting replication) and ii) younger, 5-month-old infants as different given developmental differences in face preference between 7 and 5-month-old infants [14]. Chi-Square test was used for comparing two groups in a contingency table. The analyses provided a preliminary test of whether the obtained distributions of MD scores could be used to compare an individual’s data to an a priori defined control distribution. As discussed later in the chapter “Barrier to entry…”, however, the present results cannot be regarded as genuine normative data.

#### Data availability

The data used in the current analyses are available from the authors upon request.

## Results

### Descriptive results and analyses of initial gaze disengagement

Inspection of the gaze data in the primary sample revealed two distinct time windows that reflect the main components of infants’ stereotypic gaze pattern during the trial: i) gaze fixation to the face followed by transition to the peripheral target, and ii) possible return of the gaze back to face. Traditionally, studies using the overlap paradigm in infants have focused on measuring the frequency and latency of the first gaze transition to the peripheral target as the primary dependent variable [13], [22], [25]. Analyses of these transitions in our primary sample (see figure S3 and table S1) showed that a rapid saccade from the central to the lateral stimulus (M = 388 ms, SD = 72 ms) was observed on the majority of trials, although the proportion of missing saccades varied substantially between individuals (range 6–78%, M = 44%, SD = 22%). The results also showed the predicted effect of facial expression on disengagement probability, χ2 = 15.1, df = 3, *p*<0.01. Consistent with prior studies [13], the proportion of missing saccades was lowest in the non-face control condition (M = 27%, SD = 23%), intermediate in the neutral (M = 42%, SD = 26%) and happy (M = 47%, SD = 28%) conditions, and highest in the fearful (M = 58%, SD = 27%), although it is noteworthy that none of the pairwise conditions were significant after correction for multiple testing in this small sample of 7-month-old infants (N = 13). Disengagement times (DT) varied significantly by facial expression condition, χ2 = 8.4, df = 3, *p*<0.05, with faster DT in the control condition (M = 343 ms, SD = 90 ms) than in face conditions (Neutral, M = 424 ms, SD = 89; Happy, M = 396 ms, SD = 82 ms; Fearful M = 403 ms, SD = 78 ms). Yet again none of the pairwise comparisons was significant after correction for multiple testing.

### Novel dynamic response (DR)

Inspecting the DR distribution from all infants, we found that in 86.3% of all trials (where gaze had initially shifted to the target), the infants looked back from the target to the face, and the gaze was variably alternating between the face and the target. This is clearly reflected in the shape of DR distributions in the DT-locked DR traces (figures 3A,B), and it gave us the motivation to subsequently build an index that reflects the cumulative deviation of gaze from a normative range.

In order to better understand the gaze changes within the trial time window, and to potentially use that information in infant testing, we calculated distributions of DR traces over time. There were striking time-varying differences in the shapes and scales of distribution of DR traces across the time course of the trial (figures 3A,B). For example, the distance between the 10^th^ percentile and the median DR ranged from close to zero in the beginning of the trial up to 0.3 later on (figure 3B). This encouraged us to bind the mean deviance metric to dynamically evolving percentiles rather than some a priori defined limit. The DR distributions remained stable also at the end of the trial, however the number of trials contributing to the DR average was somewhat reduced due to DT time-locking. We hence extended the time window for MD calculations from 0 ms to 2200 ms (from the disengagement) that covers all later phases of the trial.

The DR traces varied slightly between the stimuli, being generally lower for the non-face control stimulus than to faces (especially faces displaying a fearful expression, figure 3C). We then compared the stimulus-specific Mean Deviances (MD) computed from the DR traces. The MDs computed from the 13 infants (dataset from Helsinki) is presented in figure 3D, and it shows that non-face stimulus (noise) yields significantly lower MDs compared to the combined face condition (*p* = 0.05; Mann-Whitney *U* test). No significant differences were found between facial expressions, which may be partly explained by the limited numbers. These results are consistent with prior studies [13] and the a priori hypothesis of differential patterns of gaze behavior for non-face control stimuli and faces, and motivated us to include only trials with face stimuli in the subsequent analyses.

### Replication in an independent sample

**Figure 4:**
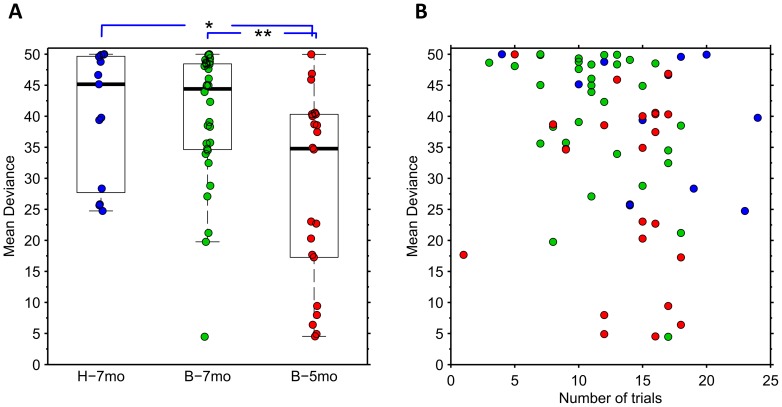
Individual and group level findings of the MD scores. A) Summary of MD findings in all three infant groups: 7-month-old Helsinki (H-7mo) and Boston infants (B-7mo), and 5-month-old Boston infants (B-5mo). Scores of individual infants’ are illustrated with coloured circles with the underlying boxplot depicting the median and IQR, and the whiskers showing the total score extent neglecting the outliers (max length 1.5*IQR from the box edge).Comparison of MD scores between younger and older infants revealed age-related differences (** and * indicate p-values under 0.02 and 0.01 respectively; Mann–Whitney U test). B) Comparison of MD score and the number of trials available from each infant shows no systematic change in MD with an increase in trial numbers. The colour coding of circles depicts individual scores as shown in figure A.

In order to assess the replicability of our measures for the classification of individual infants, we used an independently collected infant sample of same age from the laboratory in Boston (see Dataset S1 for details). These infants were found to be comparable to the infant cohort from Helsinki based on observing no significant differences in the conventionally studied measures, the mean DT and DP (all *p*-values >0.25; Mann-Whitney *U* test). Likewise, the median and the overall distribution of MD values were very compatible between the two datasets as there were no statistical differences between the groups (figure 4A). The dataset from Boston had one “outlier” participant with markedly lower MD values. Based on study logbooks, we are confident that the low MD value was not due to technical factors from the recording session or the analysis, or limited number of trials included in the MD calculation (n = 17 in figure 4B). Despite of this outlier, the group level variance (expressed as IQR in the graph) was smaller in the Boston dataset, which may be readily explained by chance effects in small datasets.

### Sensitivity to age-specific differences

Previous research has shown that preference to faces is strengthened between 6 and 9 months of age (e.g. [2]). We used this prior knowledge to test the potential of our MD metrics to detect known developmental changes in infant’s face processing. Our premise was that a reasonably sensitive test should distinguish younger (5-month-old) infants from the older (7-month-old) infants. Our current dataset is obviously too limited to provide definitive normal ranges and thereby to estimate sensitivity/specificity figures. However, the findings in figure 4A clearly show how MD values in a large proportion of the younger infants fall below the range of the older infants. For demonstration purposes, we might choose an arbitrary MD threshold, e.g. 24, at the lower bound of the MD distribution in 7-monts-old infants. Using this threshold, three (7%) out of 45 older (7 months; data from both laboratories combined) infants and ten out of 22 younger (5 months) infants would be considered deviant. This difference between groups was significant (*p*<0.05; Chi-Square test). Closer inspection of the MD results in younger infants suggests that they may fall into two subgroups, one with MD value comparable to the older group, and the other with MD value clearly below them. It will be a subject of further study to determine the neurodevelopmental correlates of these subgroups, yet the data suggests intriguing possibility of distinct developmental trajectories at this age. Taken together, the current results lend support for the idea that the relative developmental stage of infant’s visual attention could be assessed in an objective and automated manner by using MD-based metrics from eye tracking time series.

### Stability of the metrics

We then wanted to assess how much the performance of infants, as well as our eye tracking –based metrics vary within a single study session. Theoretically thinking, it is possible that the infant performance changes systematically due to e.g. exhaustion, habituation, or contingency learning. A systematic change can be readily estimated by looking at the change in the metric as a function of trial number within the session, which we computed over all subjects pooled together (figure S4A-C). We employed Sign test on split half data (first vs. last half of the trials) to analyze whether the direction of change is similar, and hence predictable across the infant group (figure S4D-F). This was motivated by the idea that finding a consistent direction of change would give a possibility to device a correction mechanism in future analyses. Finally, we also analyzed whether splitting the individual datasets into odd and even trials would give subsets that are statistically comparable. Computing the linear correlation between odd and even values shows whether our paradigm can be considered stable enough for presenting multiple conditions that are equally dispersed throughout the study session.

The possibility that child’s performance changes systematically during a test session was estimated from the disengagement probability analysis. After pooling all data together, we found that DPs decline as a function of trial number (figure S4A; r = -0.79, *p*<10∧-7; Pearson). Moreover, DPs were significantly higher in the first half compared to the last half (figure S4D; *p*<10∧-6; n = 66). Correlation between Odd and Even trials was strong (r = 0.77, *p*<10∧-13). Our observations further suggested that infants’ tendency to reduce gaze shifts during the test session might be linked to the disengagement times at individual level. When the DTs of the seven infants whose DP values declined the most across the session were compared to the seven infants with weakest DP decline a clear difference was observed; mean DTs being 461 ms (SD 61 ms) and 370 ms (SD 70 ms), respectively.

The possibility of a systematic change during a test session was then assessed separately for disengagement times and mean deviations. There was a significant decline in mean DTs during the course of study session (figure S4B; r = -0.74, *p*<10∧-5), however the Sign test between first and second half of the trials was nonsignificant (*p* = 0.18; n = 28) indicating that there is no consistent direction of change at individual level (figure S4E). There was also a strong correlation between Odd and Even trials (r = 0.63, *p*<0.001).

Also MD values tend to decrease as a function of trial number although the correlation is weaker compared to DP and DT analyses above (figure S4C; r = -0.38, *p* = 0.03). Sign test between first and second half of experiments was nonsignificant (*p* = 1; n = 28) as well, indicating that the direction of MD change during the trial was variable at individual level (figure S4F). There was also a strong correlation between Odd and Even trials (r = 0.82, *p*<10∧-7). Moreover, the MD metric seems robust in terms of individual variation in the number of trials included in the DR calculation with figure 4B presenting MD values as a function of number of trials failing to reveal apparent trends within any participant group.

## Discussion

The present results are compatible with previous findings in showing lowered probability of gaze disengagements from faces and, particularly, faces displaying a fearful expression (see [14], [28] for further discussion of this finding). The main purpose of our study was to extend these analyses by systematically characterizing infants gaze behavior after the initial gaze shift (i.e. disengagement) from face, by devising novel metrics to quantify this behavior, and by demonstrating how these metrics vary by stimulus condition (emotion) and age. In the following, we discuss differences between the conventional attention disengagement and the new metrics reflecting attentional re-engagement processes, and evaluate the potential suitability of each of these metrics for prospective use in normative studies and assessments of individual infants.

### Measures for attention and face preference

The main novel contribution in our work was related to characterizing infant’s visual exploration during the latter half of the trial period, when both the face and target stimuli are available for viewing. To parameterize these data, we used dynamic response analysis to create a metric (MD) that reflects infants’ cumulative allocation of attention to the central stimulus (as opposed to lateral geometric shape). We believe that this metric may differ from the conventional disengagement both in terms of its statistical properties and also by tapping different attentional systems.

Specifically, instead of resorting to a single numerical value of gaze shift latencies (cf. DT above), we reasoned that this time interval within the trial is about infant’s preferential choices in a more dynamic manner. Conceivably, the situation compares to the traditional sustained attention paradigms that assess infants’ capacity to resume and sustain attention with a primary stimulus after momentary distraction (e.g. [29]). The situation is also analogous to common behavioral testing where the experimenter, such as psychologist or pediatrician, is estimating the overall pattern of infant’s behavior rather than any particular point in time. Technically speaking, examiner’s perception of normality is essentially a time integral of infant’s behavior, and its translation to our eye tracking paradigm would be the temporally cumulating deviation of visual exploration (i.e., gaze) from the expected range. Based on this rationale, we devised the measure MD that was calculated in relation to the time-varying distribution of the eye tracking data in our sample of typically developing infants. The obvious advantages of this approach are that i) it is insensitive to single outlier values in each trial (removed by median filter of the gaze tracking data and averaging over multiple trials), ii) it can be adjusted to allow detection of even marginal abnormalities (by changing the threshold), and iii) it is able to measure cognitive operation (gaze attention) over extended period of time instead of single time point like the conventional DT.

As for the underlying neurocognitive processes and attentional systems, it can be speculated that the initial disengagement of gaze from the face to the lateral stimulus (DT in our analysis), and subsequent (prolonged) re-engagement with the face (MD in our analyses) reflect partially different aspects of attention. For example, the initial disengagement may be based on subcortical systems important for reflexive orienting responses in infants [15], [30] whereas the latter may engage more voluntary and goal-directed attentional control mechanisms, centered in the dorsal parietal and frontal cortex [30], [31]. Clearly, further research is needed to tease apart between these explanations, but it is interesting to note that our results showed strikingly similar MD score in two independent sample of 7 month-old infants, and a clearly differentiated these two groups from younger, 5-month-old infants. Moreover, our observation (figure 4) suggests that MD may even be able to identify different developmental subgroups in the young infants that are just approaching the age when the tested behavior is developing. Our present data, however, is not able to confirm this, or identify its wider correlates, yet the results suggest that MD might be sensitive to developmental differences.

### Barriers to entry into larger scale field studies or clinical diagnostics

Our work developed such quantifiable metrics of the face-distractor competition paradigm that could be carried further to eventually become research tools in larger scale developmental studies or even clinical screening. While we find the overall concept applicable to such use, we can identify multiple potential or actual barriers to entry, which need further attention:

#### Suitability of disengagement paradigm as a general measure of individual’s cognition

The genuine value of clinical diagnostic method comes from its performance at individual level, and such accuracy would also greatly benefit methods used in larger scale population studies. Prior works in both infant and adults populations have shown how disengagement paradigm can identify and predict abnormal neurocognitive function at the group level [32], [33], however there is a shortage of experience from the use of this (or any comparable) paradigms at the individual level assessment. Indeed, a major obstacle in obtaining such experience comes from the challenges related to conventional analyses, and we trust that the quantifiable, already partly automated analysis methods presented in our work could facilitate future prospective studies needed to provide evidence about how the study paradigms may perform at individual level.

#### Practical considerations of disengagement paradigm setup in studying infants

Bringing a test paradigm from basic science laboratory to field testing, or testing on compromised infants, requires that all parts of the paradigm ranging from the physical recording set up to time constraints and analysis pathways are compatible with often suboptimal conditions. The physical recording setup used in our work consists of an eye tracker device integrated to the computer screen, which can be readily mounted on any office table (see figure 1). We are now piloting with even lighter design where the eye tracker device is a light portable bar that can be attached on the top of any computer screen. Given that the study procedures were automated, running this system requires less than an hour training from people with average computer skills. The cost of eye tracking devices has been an issue, but recent development of consumer applications for eye tracking technology has already dropped the pricing to the level of any smaller medical devices. Another major issue is the time needed for testing, which in the infants is limited by their short attention span and vigilance state cycles. Our work showed, in full agreement with prior studies, that the group of healthy 7-month-old infants demonstrated a consistent pattern in the dynamic attentional response, and they can be readily studied from individual infants using only relatively few trials. It was hence possible to run the full test procedure within a few minutes, which we find very tolerable for practical settings.

The remaining practical consideration relates to data analysis, which in this study needed special signal analysis expertise. The present work was to create metrics that can be implemented as an automated script, and we believe that the most plausible implementation in larger scale studies would come via development of scripts that immediately output the analyses in the end of the test session.

It is notable in this context that our present work was carried out in a specialized hospital clinic (Helsinki) or in a science laboratory (Boston). Hence future studies will be needed to gain experience from running the paradigm in less equipped environment and by less skilled people. To this end, we are currently piloting this paradigm as a fully automated setup (from running the trials to its full analysis) in a basic health care facility.

#### Potential needs to improve or simplify the paradigm

The utility and power of a paradigm in any larger scale use depends partly on how well it is optimized for the task. Regarding the choice of stimuli, we showed that our metrics indicated expected differences between genuine face vs. non-face stimuli (cf. [27], [34]–[38]), and the fearful face was most distinctive of different facial expressions (see also [13], [22], [25], [26]). These observations suggest that the paradigm could be simplified to consist of one facial expression only to be combined with non-face in order to offer the most robust estimate of infant’s preference to social relevance of the stimulus (cf. [23], [27], [39]). Regarding the geometrically shaped targets used in this work, we believe that they could be made more attractive (e.g. simple animations) that would increase the frequency of gaze shifts. The practical advantage of this would be to reduce heterogeneity in the proportion of initial gaze shifts and increase the number of accepted trials, i.e. the statistical robustness of the study session. Modifications of this kind are readily implemented in the stimulus settings and they won’t imply any change in the analysis paradigm, yet they hold promise for expediting the study procedure and improving the statistical validity of the findings.

#### Challenges in validation of the study paradigm

Novel methods are validated by providing evidence of their utility for the given use. One key measure is the “accuracy” of the new method, which would be conventionally evaluated by measuring its sensitivity and specificity in detecting a given feature of cognition. While such approach would be an obvious aim for our present paradigm as well, it is important to note that formal assessment of sensitivity and specificity measures do require as yet non-existent, reliable and quantifiable criterion variable or “ground truths” of the feature of interest. Indeed, the lack of such ground truths in neurocognitive assessment was a major underlying driver for our present work. The genuine validation of this and other future tests of core cognitive functions in infants (e.g. [40]) will thus come from the test of time: the ability i) to outperform conventional behavioral assessments in study cohorts, and ii) to provide a perceived added value to studies seeking early developmental biomarkers (cf. [41]–[43]). Validation in this way will be based on larger scale recruitments of both typically and atypically developing infants, which is already running in our and other laboratories.

#### Creation of reference (normative) data

Individual level assessment is based on comparing the findings with normative data. Our present study focused on testing the repeatability of our metrics in an independent dataset, as well as the ability of the metric to detect age-related differences between older and younger infants. However, these cohorts were not suitable for establishing normative values, because the datasets were too small and we did not have complete enough information about the ultimate neurocognitive development of these babies. While we found our pilot observations encouraging, we acknowledge that future prospective studies with standardized cohort characterization will be needed to establish genuine normative values and their developmental trajectories.

### Conclusions

Our present work showed that novel analysis of eye tracking data during the face-distractor competition paradigm reveals sustained attention for faces in infants, and are sensitive to known developmental changes in infants’ face processing between 5 and 7 months of age. Our work further showed that the test paradigm and data analysis can be implemented in a standardized and automated manner outside of neurocognitive laboratories, such as a well-equipped pediatric hospital clinic. Together with a multitude of prior studies [5], [27], [32], [44], our results call for exploiting eye tracking based paradigm(s) as a potential candidate for studying neurocognitive development of individual infants at the age when conventional neuropsychological testing is challenging. Development of early biomarkers of cognition holds promise for disclosing elusive causal relationships in cognitive development, which is crucial to better understand the environmental (epigenetic) effects on cognition, pathophysiological mechanisms underlying neurocognitive morbidities [45], or even guide development of very early and targeted neurocognitive therapies [8].

## Supporting Information

Figure S1 **Calibration of the eye tracking system.** Example screenshot from a calibration session demonstrating how Tobii Studio software shows the offset of gaze during calibration procedure.
(TIF)

Figure S2 **Assessment of the practical spatial accuracy of Tobii eye tracking system.** The figure shows combined results of all 25 trials on practical spatial accuracy of our eye tracking system. The three red circles are the fixation targets, blue crosses show the actual measured point of gaze data while watching the fixation dots, and the black stippled lines depict 51 pixel margins around each dot that included 99.9% of gaze tracking. Green circles represent the average point of gaze at each target.
(TIF)

Figure S3 **Supplementary results for DT and DP analysis.** A-B) The boxplot graphs on the left indicates gaze shifts probabilities in face and non-face (noise) stimulus conditions. The initial gaze shift from the face to the target was significantly more frequent (p = 0.03; Mann-Whitney U test) when the infant was presented with the non-face as opposed to the face stimulus (medians 88% and 46%, respectively). No such difference was found between the conditions when the returning (from target to face) gaze shifts were studied. There was also no significant difference in the mean DTs between the face and the noise condition (medians were 413 ms and 320 ms, respectively). C-E) Distribution of DTs across trials (C) shows that the distribution is strongly skewed with a long tail extending to the right (note, only those trials were used where infant’s gaze eventually turned to the target). Comparisons of infants with different numbers of successful trials show that the mean and the standard deviation of DTs are relatively insensitive to the number of trials (D and E, respectively).
(TIF)

Figure S4 **Stability of the metrics.** A-C) The three graphs present scores for DP, DT and MD depicted as function of trial number within the session, which were computed over all subjects (N = 67, including the Boston datasets) pooled together. These trial-by-trial analyses reveal systematic changes in metrics during course of eye tracking session. D-E) The graphs present split half analyses for DP, DT and MD metrics to illustrate the direction of possible changes during the time course of the eye tracking session at the individual level. Each participant is presented with one line that connects the measure of the first half to the second half. Note how the direction of change is highly variable. For the DT and MD graphs, we only used participants with 12 or more scorable trials (N = 28).
(TIF)

Dataset S1 **Description of the independent sample from Boston Children’s Hospital.** To test the developed metrics, we analyzed the data from an additional sample of 7-month-old (N = 32) and 5-month-old infants (N = 22) who had participated in an independent study in Boston Children’s Hospital.
(DOC)

Information S1 **Calibration of the eye tracking system.** Description of the calibration procedure of the eye tracking system performed at the beginning of each study session using the workflow within the Tobii Studio software. See also figure S1.
(DOC)

Information S2 **Assessment of the practical spatial accuracy of Tobii eye tracking system.** Description of the simple accuracy test that was performed to evaluate the practical spatial accuracy of the eye tracking system. See also figure S2.
(DOC)

Video S1 **Example video of typical progression of consecutive trials.** The short video clip illustrates how infant’s gaze typically moves across the screen during four trials. Here the tracked gaze path is visualized by red circles overlaying the stimulus images. The growth of the circles represents the durations of successive fixations. The video was compiled using Tobii Studio software that in our case permits only use of static images. Thus, the fixation cue at the very beginning of each trial is static in the video as opposed to the animations used in our actual experiment designed using E-Prime.
(AVI)

Table S1 **Disengagement probability and time in each participant group.** The tables contain group level results of the conventional eye tracking measures in the face-distractor competition paradigm (DP and DT) that are presented for each participant group and each stimulus condition separately.
(DOC)
