# Supplementary figures and images for: Dynamic Eye Tracking Based Metrics for Infant Gaze Patterns in the Face-Distractor Competition Paradigm
Source: PLoS One. 2014 May 20;9(5):e97299. doi: 10.1371/journal.pone.0097299 (PMC4028213; doi:10.1371/journal.pone.0097299)

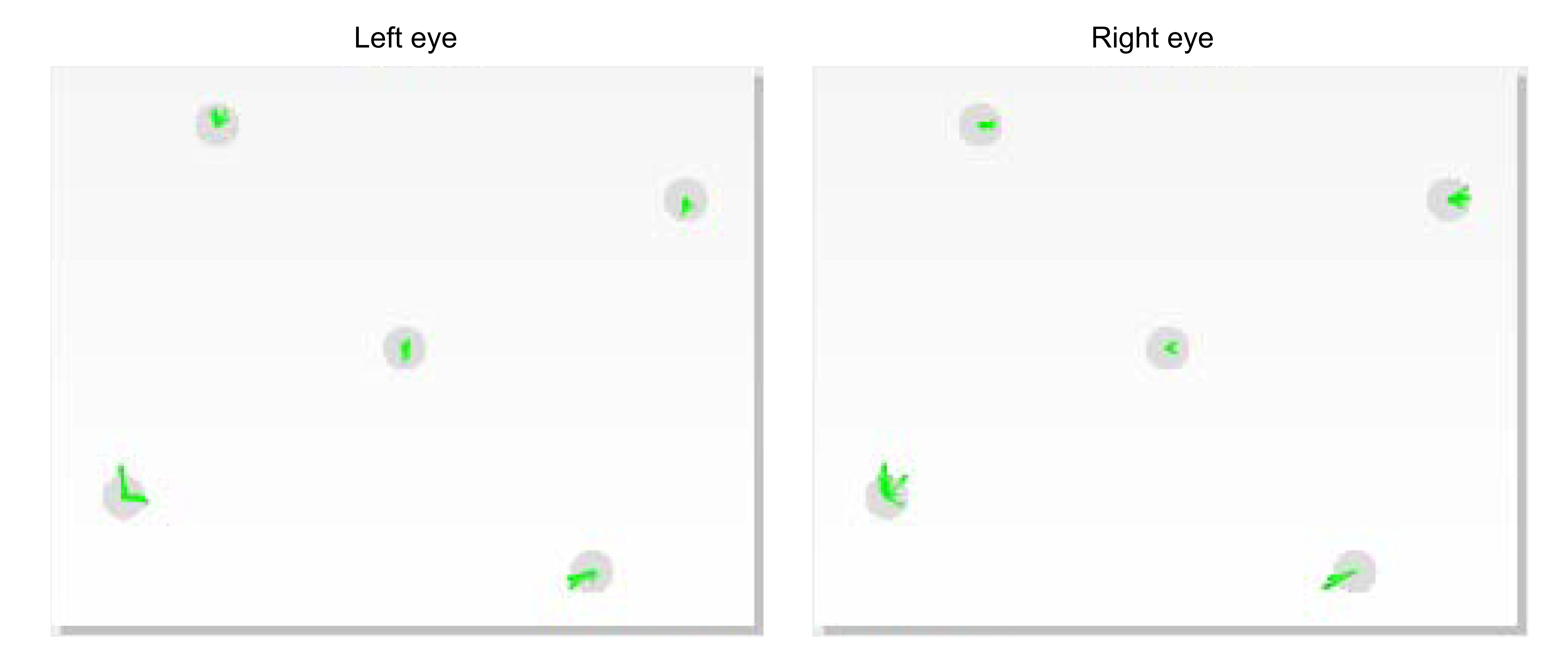

Supplement: Figure S1 — Calibration of the eye tracking system. Example screenshot from a calibration session demonstrating how Tobii Studio software shows the offset of gaze during calibration procedure. (TIF) [file pone.0097299.s001.tif]

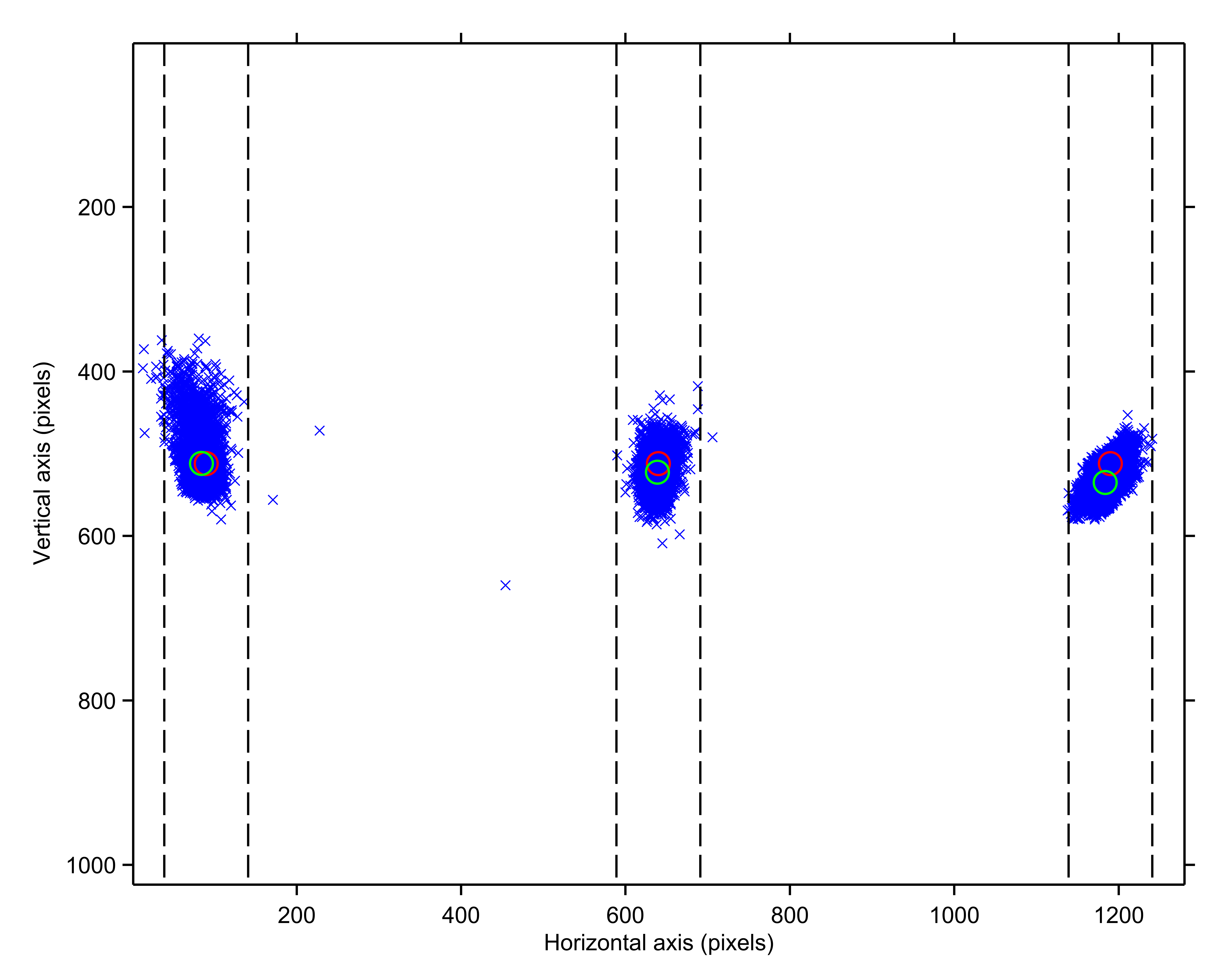

Supplement: Figure S2 — Assessment of the practical spatial accuracy of Tobii eye tracking system. The figure shows combined results of all 25 trials on practical spatial accuracy of our eye tracking system. The three red circles are the fixation targets, blue crosses show the actual measured point of gaze data while watching the fixation dots, and the black stippled lines depict 51 pixel margins around each dot that included 99.9% of gaze tracking. Green circles represent the average point of gaze at each target. (TIF) [file pone.0097299.s002.tif]
