# Supplementary material for: Dynamic Eye Tracking Based Metrics for Infant Gaze Patterns in the Face-Distractor Competition Paradigm
Source: PLoS One. 2014 May 20;9(5):e97299. doi: 10.1371/journal.pone.0097299 (PMC4028213; doi:10.1371/journal.pone.0097299)
Supplement: Dataset S1 — Description of the independent sample from Boston Children’s Hospital. To test the developed metrics, we analyzed the data from an additional sample of 7-month-old (N = 32) and 5-month-old infants (N = 22) who had participated in an independent study in Boston Children’s Hospital. (DOC) [file pone.0097299.s005.doc]

**Supplementary Dataset S1 Description of the independent sample from Boston Children’s Hospital.** To test the metrics developed in the analyses of our dataset from Helsinki University Hospital, we analyzed data from an additional sample of 7-month-old infants (N=32; age range 6.6-7.6 months, mean 7.0 months) and 5-month-old infants (N=22; age range 4.7-5.6 months, mean 5.1 months) who had participated in an independent study in the Developmental Medicine Center, Boston Children’s Hospital, and had analyzable eye tracking data available. Data from an additional three infants in the Boston sample were excluded from all analyses due to a procedural error (n=1), sleepiness (n = 1), or near absence of predicted saccades to the target (n=1). The procedure and stimuli in the Boston study were identical to the ones used in our study in Helsinki with the following exceptions: i) the testing session consisted of a total of 24 instead of 32 trials (6/condition), ii) the stimuli were neutral, happy, angry (removed from the analysis due to comparability), and fearful facial expressions, and the phase-scrambled face was not included, iii) eye tracking data were recorded at a 60 Hz instead of 120 Hz sampling rate (Tobii T60 model), and iv) screen resolution of the display was 1024 x 768 pixels (presenting the stimuli on the screen slightly larger).
