# Supplementary material for: Dynamic Eye Tracking Based Metrics for Infant Gaze Patterns in the Face-Distractor Competition Paradigm
Source: PLoS One. 2014 May 20;9(5):e97299. doi: 10.1371/journal.pone.0097299 (PMC4028213; doi:10.1371/journal.pone.0097299)
Supplement: Information S1 — Calibration of the eye tracking system. Description of the calibration procedure of the eye tracking system performed at the beginning of each study session using the workflow within the Tobii Studio software. See also figure S1. (DOC) [file pone.0097299.s006.doc]

**Supplementary Information S1. Calibration of the eye tracking system.** Each study session began with the calibration of the eye tracking system following the semi-automated workflow within the Tobii Studio software. Calibration aims to measure the characteristics of the subject’s eyes and integrate them with an internal, physiological 3D eye model, thereby improving tracking accuracy. The protocol proceeded by showing the infant an audiovisual animation sequentially in five locations on the screen. The outcome of the calibration procedure was read from a qualitative output showing the offset between measured gaze points and the center of the given calibration spot (see figure S1). Hence, the final acceptance of calibration was based on subjective assessment by the examiner. Since our present paradigm did not rely on tracking accuracy at millimeter level, we found it most practical to accept calibrations which were able to track all 10 calibration points (5 for each eye). If one or more marks were missing completely, we repeated the calibration until at least nine marks were obtained. Calibration in this way consistently gave sufficient tracking accuracy for the purposes of the present study. Only one infant had to be excluded due to an unsuccessful calibration.
