# Supplementary material for: Dynamic Eye Tracking Based Metrics for Infant Gaze Patterns in the Face-Distractor Competition Paradigm
Source: PLoS One. 2014 May 20;9(5):e97299. doi: 10.1371/journal.pone.0097299 (PMC4028213; doi:10.1371/journal.pone.0097299)
Supplement: Information S2 — Assessment of the practical spatial accuracy of Tobii eye tracking system. Description of the simple accuracy test that was performed to evaluate the practical spatial accuracy of the eye tracking system. See also figure S2. (DOC) [file pone.0097299.s007.doc]

**Supplementary Information S2. Assessment of the practical spatial accuracy of Tobii eye tracking system.** The manufacturer of our eye tracker (Tobii) promises tracking accuracy at below centimeter range, which would be sufficient for our present experimental design. However, in order to assess the clinical feasibility of testing of this kind, we performed a simple accuracy test with an adult subject. First, we completed the normal calibration procedure (see above), then showed a fixation dot in three locations on the screen for 4 sec in each location. The locations were one in the middle and one on the left and right side, 7% from the sides of the screen corresponding to the AOIs in the disengagement paradigm (see Fig. S2). The whole test was repeated beginning from routine calibration for altogether 25 times. We then analyzed the gaze data of the last three seconds (out of the 4sec) of each fixation comparing the gaze coordinates to the actual locations of the presented dots. We considered the outcome of this test to be the “practical accuracy” in realistic conditions. That information is useful in our search for AOI marginal and their tolerance.

As shown in figure S2, the gaze data during the fixation was only a very little scattered around the fixation dots. In the horizontal axis, especially, practically every (99.9 %) gaze coordinate was found within 51 pixels of the fixation dots (limits shown with stippled lines).As this data is based on a fully co-operating adult subject and does not take into account any intersubject variability, we decided to go with distinctly wider margins in our present study with less co-operating infants. However, the results indicate that the practical accuracy of our system is consistent with the specifications of the manufacturer.
