# Supplementary material for: Dynamic Eye Tracking Based Metrics for Infant Gaze Patterns in the Face-Distractor Competition Paradigm
Source: PLoS One. 2014 May 20;9(5):e97299. doi: 10.1371/journal.pone.0097299 (PMC4028213; doi:10.1371/journal.pone.0097299)
Supplement: Table S1 — Disengagement probability and time in each participant group. The tables contain group level results of the conventional eye tracking measures in the face-distractor competition paradigm (DP and DT) that are presented for each participant group and each stimulus condition separately. (DOC) [file pone.0097299.s009.doc]

**Table S1A. Disengagement probability (%) in each participant group**

| **Group** | **All** | **Face** | **Non-face** | **Happy** | **Fear** | **Neutral** | **Angry** |
| --- | --- | --- | --- | --- | --- | --- | --- |
| H7 | 56 (±22) | 51 (±24) | 73 (±23) | 53 (±28) | 42 (±27) | 58 (±26) | - |
| B7 | 51 (±23) | 51 (±24) | - | 55 (±29) | 47 (±28) | 49 (±28) | 50 (±27) |
| B5 | 66 (±26) | 68 (±25) | - | 67 (±33) | 70 (±26) | 66 (±29) | 61 (±34) |

**Table S1B. Disengagement time (ms) in each participant group**

| **Group** | **All** | **Face** | **Non-face** | **Happy** | **Fear** | **Neutral** | **Angry** |
| --- | --- | --- | --- | --- | --- | --- | --- |
| H7 | 388 (±72) | 412 (±74) | 343 (±90) | 396 (±82) | 403 (±78) | 424 (±89) | - |
| B7 | 431 (±86) | 430 (±82) | - | 438 (±106) | 418 (±79) | 441 (±115) | 433 (±135) |
| B5 | 429 (±79) | 435 (±89) | - | 459 (±93) | 437 (±116) | 414 (±110) | 405 (±84) |

Disengagement probability (DP) is calculated as the proportion of the trials with acceptable gaze shift towards the target from the total number of trials excluding the ones with either technically unreliable tracking or anticipatory gaze movement (DT <150ms). Disengagement time (DT) is the mean of the latencies from the target onset to the time when the infant shifts gaze from the face to the target (trials with latency >1000 ms excluded). The results in the tables are expressed as group means (±SDs) for the two separate groups of 7-month-olds (H7 and B7) and the one group of 5-month-old infants (B5) for each stimulus presentation separately. The joint face condition is a combination of trials with either happy, fearful or neutral stimulus. All infants with ≥2 scorable trials per condition were retained in the DT analysis (N_H7_=10, N_B7_=15, N_B5_=16) and all infants with ≥3 scorable trials per condition in the DP analysis (N_H7_=13, N_B7_=32, N_B5_=22).

The results indicate that while the DPs are comparable between the cohorts of 7-month-olds from Helsinki and Boston the younger infants in Boston generally produce higher DP values i.e. have higher tendency for the disengagement saccades. In terms of DTs the groups are very comparable, although the control condition included only in Helsinki measurements yields remarkably faster DTs than real face conditions.
